# Supplementary material for: Clinical Features and Outcomes of Persistent Candidemia Caused by Candida albicans versus Non-albicans Candida Species: A Focus on Antifungal Resistance and Follow-Up Blood Cultures
Source: Microorganisms. 2023 Apr 3;11(4):928. doi: 10.3390/microorganisms11040928 (PMC10142578; doi:10.3390/microorganisms11040928)
Supplement: Supplementary file 1 [file microorganisms-11-00928-s001.zip › microorganisms-2306480-supplementary.pdf]

Article

Clinical Features and Outcomes of Persistent Candidemia Caused by *Candida albicans* versus Non-*Albicans Candida* Species: A Focus on Antifungal Resistance and Follow-Up Blood Cultures

Shiori Kitaya [1,2,*,†], Hajime Kanamori [1,*,†], Yukio Katori [2] and Koichi Tokuda [1]

[1] Department of Infectious Diseases, Internal Medicine, Graduate School of Medicine, Tohoku University, Sendai 980-8574, Japan; tokuda@med.tohoku.ac.jp

[2] Department of Otolaryngology, Head and Neck Surgery, Graduate School of Medicine, Tohoku University, Sendai 980-8574, Japan; yukio.katori.d1@tohoku.ac.jp

* Correspondence: shiori.kitaya.b7@tohoku.ac.jp (S.K.); kanamori@med.tohoku.ac.jp (H.K.)

† These authors contributed equally to this work.

Table S1. Clinical characteristics of hospital-acquired persistent candidemia in terms of azole or echinocandin resistance and clearance of persistent candidemia.

	HA-PC Resistant Strains Non-Clearance Group (n = 15)	HA-PC Resistant Strains Clearance Group (n = 19)	Odds Ratio [95% CI]	p-Value	HA-PC Susceptive Strains Non-Clearance Group (n = 6)	HA-PC Susceptive Strains Clearance Group (n = 20)	Odds Ratio [95% CI]	p-Value
**Demography**								
Sex (male, %)	6 (40.0)	13 (68.4)	0.3 [0, 1.3]		5 (83.3)	14 (70.0)	2.1 [0.2, 22.5]	
Age, years, median (IQR)	65.0 (53.0–77.0)	62.5 (52.0–68.0)		0.025	67.0 (59.5–71.5)	65.5 (48.5–75.3)		
**Comorbidities**								
Diabetes mellitus	2 (13.3)	2 (10.5)	1.3 [0.2, 10.6]		0 (0)	6 (30.0)	0	
ESDR on hemodialysis	0 (0)	0 (0)	–		0 (0)	1 (5.0)	0	
Liver cirrhosis	0 (0)	3 (15.8)	0		0 (0)	0 (0)	–	
Solid malignancy	5 (33.3)	7 (36.8)	0.9 [0.2, 3.6]		3 (50.0)	12 (60.0)	0.7 [0.1, 4.2]	
Hematologic malignancy	2 (13.3)	2 (10.5)	1.3 [0.2, 10.6]		0 (0)	0 (0)	–	
Neutropenia	3 (20.0)	2 (10.5)	2.1 [0.3, 14.7]		0 (0)	0 (0)	–	
Immunosuppression	5 (33.3)	5 (26.3)	1.4 [0.3, 6.2]		2 (33.3)	1 (5.0)	9.5 [0.7, 132]	
**Vital signs**								
BMI, kg/m², median (IQR)	18.6 (16.5–21.8)	19.8 (17.5–23.6)			18.8 (17.2–20.2)	21.0 (18.1–23.4)		
Body temperature, °C, median (IQR)	38.1 (37.4–38.5)	37.9 (37.2–38.8) (n = 16)			38.6 (36.9–39.3) (n = 5)	39.0 (38.6–39.6) (n = 18)		
**Laboratory markers**								
White blood cell count, 10⁹/L, median (IQR)	6,300.0 (2,550.0–10,850.0)	6,400.0 (3,900.0–11,550.0)			18,500.0 (9,375.0–22,525.0)	9,400.0 (7,050.0–13,225.0)		
Neutrophil count, 10⁹/L, median (IQR)	5,950.0 (1,545.0–9,555.0)	4,430.0 (2,835.0–10,305.0)			17,405.0 (7,752.5–20,615.0)	7,390.0 (6,070.0–11,590.0) (n = 19)		
C-reactive protein, mg/dL, median (IQR)	10.8 (7.0–13.0)	6.3 (1.5–10.6)			8.4 (6.3–10.7)	8.3 (4.6–10.3)		
**Devices**								
Intravascular device	14 (93.3)	16 (84.2)	2.6 [0.2, 28.2]		4 (66.7)	14 (70.0)	0.9 [0.1, 6]	

Intravascular device removal	11 (78.6)	15 (93.8)	0.2 [0, 2.7]	3 (75.0)	13 (92.9)	0.2 [0, 4.8]
Cardiovascular surgery	2 (13.3)	4 (21.1)	0.6 [0.1, 3.7]	0 (0)	2 (10.0)	0
ECMO	2 (13.3)	1 (5.3)	2.8 [0.2, 33.9]	1 (16.7)	0 (0)	–
Continuous hemodiafiltration	4 (26.7)	4 (21.1)	1.4 [0.3, 6.7]	2 (33.3)	2 (10.0)	4.5 [0.5, 42.2]
Mechanical ventilation	3 (20.0)	6 (31.6)	0.5 [0.1, 2.7]	2 (33.3)	7 (35.0)	0.9 [0.1, 6.4]
**Status of persistent candidemia**						
The period until FUBC is carried out, median (IQR)	5.0 (2.0–6.0)	3.0 (2.5–5.0)		3.0 (2.3–4.5)	3.5 (2.0–6.3)	
Duration of candidemia, median (IQR)	7.0 (4.5–12.5)	6.0 (3.0–10.0)		4.5 (3.3–5.0)	6.5 (3.5–7.3)	
**Site of infection**						
CRBSI	15 (68.2)	12 (80.0)	0.5 [0.1, 2.5]	2 (33.3)	11 (40.7)	0.7 [0.1, 4.7]
Endovascular devices infections	2 (9.1)	0 (0)	–	0 (0)	1 (3.7)	0
Septic embolism	0 (0)	0 (0)	–	0 (0)	1 (3.7)	0
Thrombophlebitis	2 (9.1)	0 (0)	–	0 (0)	2 (7.4)	0
Infected aneurysm	0 (0)	0 (0)	–	0 (0)	1 (3.7)	0
Intraocular candidiasis	0 (0)	0 (0)	–	1 (16.7)	4 (14.8)	1.2 [0.1, 12.6]
Skin and soft tissue infections	1 (4.5)	0 (0)	–	0 (0)	0 (0)	0
Abscess	1 (4.5)	1 (6.7)	0.7 [0, 11.6]	0 (0)	1 (3.7)	0
Intra-abdominal infections	0 (0)	0 (0)	–	0 (0)	1 (3.7)	0
Urinary tract infections	0 (0)	0 (0)	–	0 (0)	1 (3.7)	0
Unknown	1 (4.5)	2 (13.3)	0.3 [0, 3.8]	3 (50.0)	4 (14.8)	5.8 [0.8, 39.2]
**Hospital stays**						
Duration of hospitalization, days, median (IQR)	58.0 (44.0–79.0)	67.0 (55.0–135.8)		63.5 (38.3–97.0)	91.0 (67.3–134.8)	
Presence of ICU	5 (33.3)	7 (36.8)	0.9 [0.2, 3.6]	4 (66.7)	11 (55.0)	1.6 [0.2, 11.1]
Duration of ICU stay, days, median (IQR)	0 (0–25.0)	0 (0–19.0)		3.5 (0.8–14.5)	0.5 (0–9.3)	
Presence of HCU	0 (0)	2 (10.5)	0	0 (0)	3 (15.0)	0

Duration of HCU stay, days, median (IQR)	0 (0–0)	0 (0–0)		0 (0–0)	0 (0–0)		
Presence of CCU	0 (0)	1 (5.3)	0	0 (0)	0 (0)	–	
Duration of CCU stay, days, median (IQR)	0 (0–0)	0 (0–0)		0 (0–0)	0 (0–0)		
**Intervention**							
The use of antibiotics (Appropriate)	15 (100)	16 (84.2)	–	6 (100)	20 (100)	–	
Source control	11 (73.3)	15 (78.9)	0.7 [0.1, 3.6]	1 (16.7)	12 (60.0)	0.1 [0, 1.4]	
**Mortality**							
Early (30-day) mortality	1 (6.7)	0 (0)	–	0 (0)	0 (0)	–	
Late (30–90-day) mortality	5 (33.3)	3 (15.8)	2.7 [0.5, 13.7]	3 (50.0)	1 (5.0)	19 [1.5, 248.2]	0.028
90-day mortality	6 (40.0)	3 (15.8)	3.6 [0.7, 17.8]	3 (50.0)	1 (5.0)	19 [1.5, 248.2]	0.028

Data are presented as numbers (%) unless indicated otherwise. In the table, *p*-values are listed only for items that show significant differences. The blood test was performed on the same day as the blood culture collection. Immunosuppression was considered in neutropenia, hematopoietic stem-cell transplantation, solid organ transplantation, and corticosteroid therapy (prednisone 16 mg per day for 15 days). Cardiovascular surgery includes valve replacement, vascular graft replacement, ventricular assist device, and cardiac device implantation. Endovascular device infections encompass those of vascular grafts and left ventricular assist devices. Hospital-acquired persistent candidemia was defined as cases in which the same *Candida* species were detected on two or more consecutive occasions from BCs collected > 48 h after admission. Resistant *Candida* strains were defined as those strains resistant or susceptible in a dose-dependent manner to one or both azole or echinocandin. BMI, body mass index; CCU, coronary care unit; CI, confidence interval; CRBSI, catheter-related bloodstream infection; ECMO, extracorporeal membrane oxygenation; ESDR, end-stage renal disease; FUBC, follow-up blood culture; HA-PC, hospital-acquired persistent candidemia; HCU, high care unit; ICU, intensive care unit; IQR, interquartile range.
